# Differential impact of serum total bilirubin level on cerebral atherosclerosis and cerebral small vessel disease

**DOI:** 10.1371/journal.pone.0173736

**Published:** 2017-03-20

**Authors:** Jonguk Kim, Seung-Jae Yoon, Min-Hee Woo, Sang-Heum Kim, Nam-Keun Kim, Jinkwon Kim, OK-Joon Kim, Seung-Hun Oh

**Affiliations:** 1 Department of Neurology, CHA Bundang Medical Center, CHA University, Seongnam, South Korea; 2 Department of Radiology, CHA Bundang Medical Center, CHA University, Seongnam, South Korea; 3 Institute for Clinical Research, CHA Bundang Medical Center, CHA University, Seongnam, South Korea; University of Missouri Health Care, UNITED STATES

## Abstract

**Background:**

A low serum total bilirubin (T-bil) level is associated with an increased risk of atherosclerosis. However, the differential impact of the serum T-bil level on cerebral atherosclerosis and cerebral small vessel disease (SVD) is still unclear.

**Methods:**

We evaluated serum T-bil levels from 1,128 neurologically healthy subjects. Indices of cerebral atherosclerosis (extracranial arterial stenosis [ECAS] and intracranial arterial stenosis [ICAS]), and indices of SVD (silent lacunar infarct [SLI], and moderate-to-severe white matter hyperintensities [msWMH]) were evaluated by the use of brain magnetic resonance imaging (MRI) and MR angiography.

**Results:**

In logistic regression analysis after adjusting for confounding variables, subjects within middle T-bil (odds ratio [OR]: 0.63; 95% CI: 0.41–0.97) and high T-bil tertiles (OR: 0.54; 95% CI: 0.33–0.86) showed a lower prevalence of ECAS than those in a low T-bil tertile. Although subjects with a high T-bil tertile had a lower prevalence of ICAS than those with a low T-bil tertile, the statistical significance was marginal after adjusting for confounding variables. There were no significant differences in the proportions of subjects with SLI and msWMH across serum T-bil tertile groups.

**Conclusions:**

The serum T-bil level is negatively associated with cerebral atherosclerosis, especially extracranial atherosclerosis, but not with SVD.

## Introduction

Ischemic stroke is a heterogeneous disease that is caused by various pathomechanisms, including cerebral atherosclerosis, cardioembolism, and small vessel disease (SVD) [1]. Atherosclerotic stenosis at the extracranial and/or intracranial cerebral arteries is usually found even in the asymptomatic cases. Cerebral SVD is also found in neurologically healthy people on brain imaging as features of silent lacunar infarct (SLI) and white matter hyperintensities (WMH). Cerebral atherosclerosis and SVD share classical cardiovascular risk factors. However, the pathomechanism is quite different between them. Cerebral SVD results from the non-atherosclerotic mechanism, such as lipohyalinosis of perforating arteries [2]. Endothelial dysfunctions and an altered blood–brain barrier play a role in the development of cerebral SVD [3, 4].

Bilirubin is formed from biliverdin by the activities of heme catabolism and biliverdin reductase. Bilirubin is recognized as a potent antioxidant, and anti-inflammatory product [5, 6]. Several clinical observations have demonstrated that a high serum bilirubin level is associated with a reduced prevalence of vascular diseases, such as coronary arterial disease [7], peripheral arterial occlusive disease [8], and carotid atherosclerosis [9–13]. In addition, recent studies reported on the negative association between lower bilirubin concentration and the incidence of cerebral SVD [14, 15]. These finding indicates that serum bilirubin level is also associated with cerebral microangiopathy of non-atherogenic origin. However, data for the association of serum bilirubin with cerebral SVD is, to date, scarce. In addition, whether either cerebral atherosclerosis or SVD is more related to serum bilirubin is unclear. In the present study, we investigated the differential impact of serum bilirubin on cerebral atherosclerosis and SVD in neurologically healthy population.

## Methods

### Study design

The study was designed as a retrospective analysis of neurologically healthy individuals who visited the outpatient clinic of the Department of Neurology or Healthcare Center in the CHA Bundang Medical Center between March 2008 and December 2014. All of the subjects presented for routine health examinations or medical attention because of underlying cardiovascular risk factors. We only included individuals with ages ranging from 50 to 79 years who underwent brain magnetic resonance imaging (MRI) and magnetic resonance (MR) angiography. We reviewed the medical records, results of laboratory tests, and radiological findings. We included only subjects whose records contained adequate demographic, radiological, and laboratory data. Of 1,273 study subjects, we excluded 145 for the following reasons: (1) inadequate medical information (n = 32); (2) no laboratory tests performed (n = 29); (3) previous history of neurological disease (n = 19); and (4) a history of liver disease, including active hepatitis, liver cirrhosis, and hepatoma (n = 65). A total of 1,128 subjects were included in this study. Each subject’s data was de-identified prior to analysis. Among them, the clinical and radiological information of 946 subjects were previously described elsewhere [16]. The Institutional Review Board (IRB) of CHA Bundang Medical Center approved the study (IRB no.: BD-2010-083).

### Evaluation of clinical characteristics

The demographic data of study subjects were gender, and a history of cardiovascular risk factors, including hypertension, diabetes mellitus, current smoking, hypercholesterolemia, and coronary artery occlusive d**i**sease (CAOD). Hypertension was diagnosed if subject had a systolic blood pressure (SBP) of ≥ 140 mmHg or a diastolic blood pressure (DBP) of ≥ 90 mmHg on repeated measurement, or the subject was on antihypertensive medication. Diabetes mellitus was diagnosed if the subject had a fasting plasma glucose ≥ 126 mg/dL or was taking antidiabetic medications or insulin. Hypercholesterolemia was diagnosed if the subject had a total cholesterol ≥ 220 mg/dL or was taking lipid-lowering agents. Current smoking was defined if the subject had smoked within one year prior to examination. CAOD was diagnosed if subjects had a history of acute myocardial infarction, unstable angina, CAOD confirmed by angiography, or coronary surgery or intervention.

### Measurement of serum total bilirubin and other laboratory parameters

After 12 hours of fasting, all blood tests were performed in the core laboratory of our hospital. Serum total bilirubin (T-bil) was measured by the Evelyn–Malloy method using a Hitachi 7600 Chemistry System Autoanalyzer (Hitachi, Tokyo, Japan). Other laboratory data collected for analysis included white blood cell counts, hematocrit, platelet counts, estimated glomerular filtration rate (eGFR), and fasting glucose, total cholesterol, triglyceride, albumin, aspartate transaminase (AST), alanine transaminase (ALT), and alkaline phosphatase (ALP) levels. The eGFR was calculated using the abbreviated Modification of Diet in Renal Disease Study Equation (186 × serum creatinine^-1.154^ × age^-0.203^ × 0.742 [if female)]) [17].

### Measurement of cerebral atherosclerosis and cerebral small vessel diseases

Brain MRI and MR angiography were performed using one of three 1.5T MR systems (Sonata, Siemens Healthcare [n = 918]; Signa Excite, GE Healthcare [n = 98]; Signa HDx, GE Healthcare [n = 112]) at subjects’ own expense. A neuroradiologist who was blinded with regard to clinical and laboratory data assessed images. We evaluated extracranial arterial stenosis (ECAS) and intracranial arterial stenosis (ICAS) as MR indices of cerebral atherosclerosis according to the location of atherosclerotic lesions visualized by MR angiography. The ECAS was defined as stenosis of 50% or more in the external cranial portion of the internal carotid artery or vertebral artery on gadolinium contrast-enhanced MR angiography, using methods described in the North American Symptomatic Carotid Endarterectomy Trial (NASCET) study [18]. The ICAS were defined as stenosis of 50% or more in the proximal portions of the middle cerebral artery, anterior cerebral artery, posterior cerebral artery, intracranial portion of the vertebral artery, and basilar artery on time-of-flight images, using the method described in the Warfarin versus Aspirin for Symptomatic Intracranial Disease study [19]. Normal arterial variations, such as a unilateral origination of the bilateral anterior cerebral arteries or fetal-type posterior cerebral artery, were not regarded as true atherosclerosis.

We next evaluated SLI and cerebral WMH as MR indices of cerebral SVD visualized on brain MRI. The SLI was defined as a small (3–15 mm in diameter) cavitary lesion in an area of low signal intensity on T1-weighted images (repetition time (TR)/echo time (TE) = 560/14 ms). The WMH was defined as a hyperintense lesion in an area of bilateral cerebral white matter visualized by a fluid attenuated inversion recovery (FLAIR) image (TR/TE = 9000/105 ms, inversion time, 2500 ms). The severity of the WMH was scored by the use of a visual grading method proposed by Fazekas et al. [20]. Scores of periventricular and deep subcortical white matters were added together (ranging from 0 to 6 points), and moderate-to-severe WMH (msWMH) was defined as a total score of the periventricular- and deep subcortical WMH was three points or more.

### Statistical analysis

To evaluate the factors associated with serum T-bil levels, subjects were divided into three groups based on serum T-bil tertile: 1) low T-bil tertile group (T1): serum T-bil level ≤ 0.45 mg/dL; 2) middle T-bil tertile group (T2): serum T-bil level ranging from 0.46 to 0.67 mg/dL; and 3) high T-bil tertile group (T3): serum T-bil level ≥ 0.68 mg/dL. A between group comparison was conducted by the use of analysis of variance (ANOVA) for continuous variables and a Chi-square test for categorical variables. To evaluate the independent association of ECAS, ICAS, SLI, or msWMH across serum T-bil tertile groups, logistic regression analyses were performed between each group with a low T-bil tertile group as a reference group. To evaluate the independent associations of each MR index of cerebral atherosclerosis (ECAS and ICAS) or SVD (SLI and msWMH) with the serum T-bil level, potential confounding factors were adjusted including age, gender, hypertension, diabetes, hypercholesterolemia, current smoking, CAOD, eGFR, and other significant variables obtained from univariate analysis. Odds ratio (OR) and 95% confidence intervals (CIs) were calculated. To better understand the effect of serum T-bil levels on dependent variables, we constructed spline curves based on generalized additive model. We divided study subjects into two groups according to the presence or absence of each MR index of cerebral atherosclerosis or SVD, and conducted logistic analysis between the two groups to evaluate independent risk factors for each MR index of cerebral atherosclerosis or SVD. The raw data for statistical analysis were presented in Supporting Information (S1 Table). All statistical analyses were performed using the R package for Windows (version 3.1.3; R Foundation for Statistical Computing, Vienna, Austria). A two-sided *p* < 0.05 was considered statistically significant.

## Results

The mean age of the 1,128 study subjects was 64.2 ± 7.7 years (range, 50–79 years), and 61.9% were women. The mean serum T-bil level was 0.62 ± 0.34 mg/dL. The prevalence of ECAS and ICAS was 13.0%, and 11.3%, respectively. The prevalence of SLI and msWMH was 14.5% and 29.6%, respectively. An analysis of the clinical characteristics of study subjects revealed that gender, current smoking, SBP, DBP, hematocrit, WBC count, platelet count, and albumin, glucose, AST, ALT, and triglyceride levels were significantly different between T-bil tertile groups (Table 1). A high serum T-bil level was likely to be associated with the male gender, current smoking, high SBP, high hematocrit, high AST, high ALT, low WBC count, low platelet count, and low triglyceride level in a Tukey B post-hoc analysis. The prevalence of ECAS was significantly different among T-bil tertile groups, with a high T-bil level associated with a low prevalence of ECAS (Table 1). The prevalence of ICAS, SLI, and msWMH did not differ among T-bil tertile groups.

**Table 1:** Clinical characteristics of study subjects according to the serum total bilirubin tertile.

	ALL (N = 1128)	T1 (N = 361)	T2 (N = 396)	T3 (N = 371)	*P*
Gender (female)	698 (61.9%)	266 (73.7%)	256 (64.6%)	176 (47.4%)	< 0.001
Age, years	64.2 ± 7.7	64.5 ± 8.0	64.3 ± 7.5	63.9 ± 7.5	0.606
Hypertension	618 (54.8%)	184 (51.0%)	220 (55.6%)	214 (57.7%)	0.176
Diabetes mellitus	245 (21.7%)	93 (25.8%)	77 (19.4%)	75 (20.2%)	0.075
Hypercholesterolemia	111 (30.7%)	141 (35.6%)	108 (29.1%)	360 (31.9%)	0.132
Current smoking	236 (20.9%)	59 (16.3%)	75 (18.9%)	102 (27.5%)	0.001
CAOD	61 (5.4%)	27 (7.5%)	19 (4.8%)	15 (4.0%)	0.097
Statin medication	255 (22.6%)	77 (21.3%)	99 (25.0%)	79 (21.3%)	0.368
SBP, mmHg	131.4 ± 17.9	129.9 ± 7.1	131.0 ± 16.7	133.3 ± 19.6	0.036
DBP, mmHg	80.2 ± 11.5	78.9 ± 11.3	80.2 ± 11.2	81.5 ± 11.9	0.010
White blood cell count, × 10^9^/L	6.6 ± 2.0	6.8 ± 2.1	6.4 ± 1.8	6.5 ± 2.1	0.035
Haematocrit, %	40.2 ± 4.0	38.9 ± 3.9	40.2 ± 3.7	41.5 ± 4.1	< 0.001
Platelet count, × 10^9^/L	233.8 ± 63.1	244.5 ± 65.3	238.0 ± 60.0	218.8 ± 61.6	< 0.001
Albumin, g/dL	4.31 ± 0.40	4.26 ± 0.42	4.33 ± 0.39	4.31 ± 0.39	0.012
Glucose, mg/dL	127.0 ± 48.2	132.6 ± 53.4	119.6 ± 39.5	129.4 ± 50.5	0.001
AST, IU/L	23.9 ± 13.0	22.9 ± 8.5	23.0 ± 7.8	25.8 ± 19.3	0.002
ALT, IU/L	23.8 ± 18.7	22.3 ± 11.8	22.6 ± 12.0	26.5 ± 27.7	0.003
ALP, IU/L	181.9 ± 59.5	185.5 ± 59.2	178.2 ± 61.3	182.5 ± 57.8	0.239
Total cholesterol, mg/dL	192.7 ± 41.7	193.3 ± 41.0	193.3 ± 44.2	191.6 ± 39.6	0.823
Triglyceride, mg/dL	152.3 ± 103.4	173.5 ± 126.4	148.8 ± 96.6	135.4 ± 79.7	< 0.001
eGFR, mL/min/1.73 m^2^	74.9 ± 17.6	73.4 ± 19.9	74.7 ± 16.7	76.4 ± 16.2	0.081
Cerebral atherosclerosis					
ECAS	147 (13.0%)	62 (17.2%)	46 (11.6%)	39 (10.5%)	0.016
ICAS	127 (11.3%)	48 (13.3%)	46 (11.6%)	33 (8.9%)	0.163
Cerebral small vessel disease					
SLI	164 (14.5%)	52 (14.4%)	51 (12.9%)	61 (16.4%)	0.374
msWMH	334 (29.6%)	114 (31.6%)	115 (29.0%)	105 (28.3%)	0.595

Values are percentages, or mean ± standard deviation. *P* values were derived from an analysis of variance across T-bil tertile groups. T1, T2, and T3 represent low, middle, and high T-bil tertile groups, respectively. SBP: systolic blood pressure, DBP: diastolic blood pressure, CAOD: coronary arterial occlusive disease, AST: aspartate transaminase, ALT: alanine transaminase, ALP: alkaline phosphatase, ECAS: extracranial arterial stenosis, ICAS: intracranial arterial stenosis, SLI: silent lacunar infarct, msWMH: moderate to severe cerebral white matter hyperintensities, eGFR, estimated glomerular filtration rate

**Fig 1:**
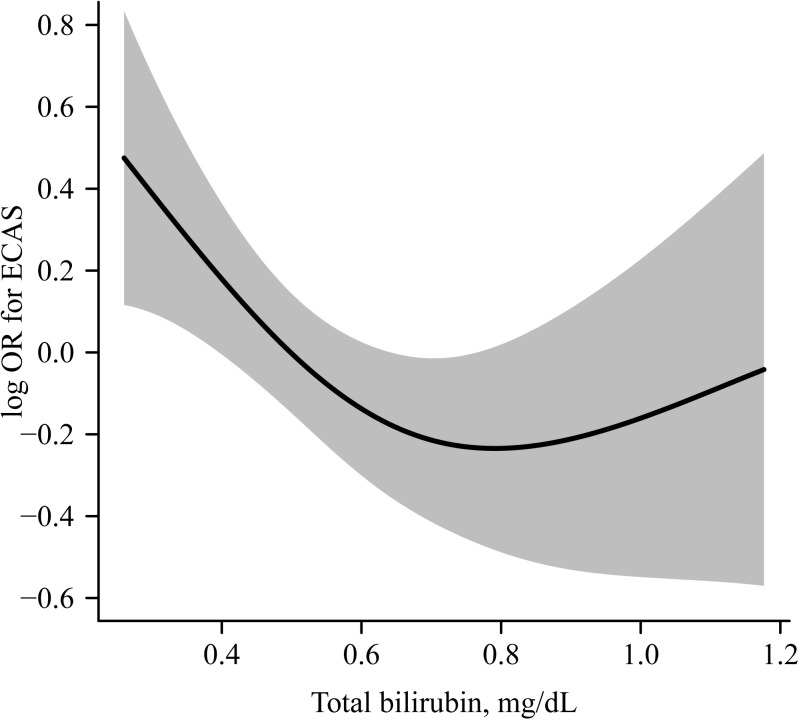
Spline curves of the relationship between T-bilirubin level and the presence of ECAS. The black lines and gray shadows represent the estimated probability and 95% confidence intervals for the presence of ECAS at the level of T-bil, and were derived from the generalized additive model. Adjustment is performed for same covariates used in model 3 of Table 2 (sex, age, hypertension, diabetes mellitus, hypercholesterolemia, current smoking, CAOD, Statin medication, eGFR, hematocrit, platelet count, albumin, AST, and triglyceride). The x-axis is limited from the 5th to 95th percentile of serum T-bil. T-bil: total bilirubin; ECAS: extracranial arterial stenosis.

We conducted logistic regression analyses to compare ECAS, ICAS, SLI, and msWMH across T-bil tertile groups (Table 2). The prevalence of ECAS was significantly lower in the middle and high T-bil tertile groups than in the low T-bil tertile group. After adjusting for cardiovascular risk factors (model 2), and for cardiovascular risk factors and other statistically significant variables obtained after univariate analysis (model 3), the statistical significance remained unchanged. In addition, such statistical significance also remained unchanged after the exclusion of eight cases with potential Gilbert syndrome (serum T-bil ≥ 2.0 mg/dL). The negative association between the presence of ECAS and T-bil level is also found in the spline curve based on generalized additive model (Fig 1). The prevalence of ICAS was significantly lower only in the high, compared to the low, T-bil tertile group factors (model 1). However, the association was marginal after adjusting for cardiovascular risk factors (model 2), and was insignificant after further adjusting for statistically significant variables obtained from univariate analysis (model 3). In the logistic regression analyses of cerebral SVD, differences were not observed for the prevalence of SLI and msWMH across serum T-bil tertiles.

**Table 2:** Logistic regression analysis of extracranial arterial stenosis, intracranial arterial stenosis, silent lacunar infarct, and moderate to severe cerebral white matter hyperintensities according to serum total bilirubin tertile.

		ECAS		ICAS		SLI		msWMH	
		OR (95% CIs)	*P* value	OR (95% CIs)	*P* value	OR (95% CIs)	*P* value	OR (95% CIs)	*P*
Model 1	T1	Ref	-	Ref	-	Ref	-	Ref	-
	T2	0.61 (0.40–0.93)	0.021	0.61 (0.54–1.31)	0.455	0.81 (0.53–1.24)	0.335	0.91 (0.64–1.02)	0.564
	T3	0.51 (0.33–0.81)	0.004	0.61 (0.37–0.98)	0.044	1.04 (0.68–1.60)	0.848	0.96 (0.67–1.37)	0.822
Model 2	T1	Ref	-	Ref	-	Ref	-	Ref	-
	T2	0.62 (0.41–0.95)	0.030	0.88 (0.56–1.39)	0.591	0.84 (0.54–1.31)	0.441	0.91 (0.64–1.28)	0.570
	T3	0.54 (0.34–0.85)	0.008	0.64 (0.39–1.05)	0.076	1.03 (0.66–1.61)	0.900	0.94 (0.65–1.35)	0.720
Model 3	T1	Ref	-	Ref	-	Ref	-	Ref	-
	T2	0.63 (0.41–0.97)	0.038	0.96 (0.60–1.53)	0.869	0.92 (0.58–1.45)	0.723	0.95 (0.67–1.36)	0.787
	T3	0.54 (0.33–0.86)	0.011	0.71 (0.42–1.20)	0.199	1.14 (0.70–1.84)	0.610	1.06 (0.71–1.57)	0.787

T1, T2, and T3 represent low, middle, and high T-bil tertile groups, respectively.

Model 1: adjusted for age and sex. Model 2: adjusted for age, sex, hypertension, diabetes mellitus, hypercholesterolemia, current smoking, CAOD, statin medication, and eGFR. Model 3: adjusted for age, sex, hypertension, diabetes mellitus, hypercholesterolemia, current smoking, CAOD, statin medication, eGFR, hematocrit, platelet count, albumin, AST, and triglycerides. Ref represents the low T-bil tertile group as a reference group for analysis. OR: odds ratio, CIs: confidence intervals, ECAS: extracranial arterial stenosis, ICAS: intracranial arterial stenosis, SLI: silent lacunar infarct, msWMH: moderate to severe cerebral white matter hyperintensities

We next conducted logistic regression analyses to evaluate the risk factors that were related to each MR index of cerebral atherosclerosis and SVD (Table 3). With adjustments for confounding factors, subjects with ECAS had a significantly lower serum T-bil level compared to those without ECAS. Other significant variables for ECAS were male gender, old age, high prevalence of diabetes mellitus, and increased AST level. The prevalence of hypercholesterolemia and statin medication was marginally higher in subjects with an ECAS than in those without an ECAS. In logistic regression analyses, serum T-bil levels in subjects with an ICAS, SLI and msWMH did not differ from those of their corresponding controls. Significant variables for ICAS were old age, and a high prevalence of hypertension and diabetes mellitus. The low eGFR was marginally significant. Significant variables for SLI were male gender, old age, a high prevalence of hypertension and diabetes mellitus, and a low eGFR. Current smoking was marginally significant. Significant variables for msWMH were old age, a high prevalence of hypertension, and a low eGFR (Table 3).

**Table 3:** Logistic regression analysis of extracranial arterial stenosis, intracranial arterial stenosis, overt white matter hyperintensities, and silent lacunar infarct.

Clinical characteristics	ECAS (n = 147)		ICAS (n = 127)		SLI (n = 159)		WMH (n = 334)	
	OR (95% CIs)	*P*	OR (95% CIs)	*P*	OR (95% CIs)	*P*	OR (95% CIs)	*P*
Gender, female	0.58 (0.38–0.88)	0.011	0.69 (0.44–1.09)	0.112	0.56 (0.37–0.85)	0.006	1.32 (0.94–1.86)	0.114
Age	1.04 (1.01–1.06)	0.006	1.05 (1.02–1.07)	0.002	1.05 (1.03–1.08)	< 0.001	1.12 (1.10–1.15)	< 0.001
Hypertension	1.32 (0.90–1.93)	0.159	1.76 (1.15–2.69)	0.009	2.39 (1.61–3.55)	< 0.001	1.52 (1.13–2.05)	0.005
Diabetes mellitus	1.95 (1.32–2.89)	0.001	1.71 (1.13–2.58)	0.012	1.62 (1.10–2.39)	0.014	1.23 (0.88–1.72)	0.218
Hypercholesterolemia	1.62 (0.98–2.69)	0.062	0.72 (0.39–1.33)	0.295	0.85 (0.50–1.45)	0.548	1.09 (0.71–1.66)	0.698
Current smoking	0.79 (0.48–1.31)	0.364	0.98 (0.58–1.65)	0.926	1.51 (0.96–2.36)	0.074	1.27 (0.86–1.88)	0.239
CAOD	1.05 (0.51–2.16)	0.887	1.28 (0.63–2.57)	0.493	0.84 (0.41–1.75)	0.645	0.77 (0.42–1.40)	0.386
Statin medication	0.58 (0.33–1.03)	0.064	1.66 (0.87–3.14)	0.123	0.85 (0.47–1.54)	0.849	0.73 (0.45–1.16)	0.184
eGFR	0.99 (0.98–1.00)	0.074	0.99 (0.98–1.01)	0.064	0.98 (0.97–0.99)	0.001	0.99 (0.98–1.00)	0.029
AST	1.01 (1.00–1.02)	0.036	1.00 (0.98–1.01)	0.660	1.00 (0.98–1.01)	0.603	1.01 (1.00–1.02)	0.194
T-bil	0.47 (0.24–0.94)	0.033	0.57 (0.28–1.16)	0.118	0.86 (0.49–1.51)	0.610	0.87 (0.56–1.37)	0.549

CAOD: coronary arterial occlusive disease, eGFR, estimated glomerular filtration rate, AST: aspartate transaminase, T-bil: serum total bilirubin, OR: odds ratio, CIs: confidence intervals, ECAS: extracranial arterial stenosis, ICAS: intracranial arterial stenosis, WMH: white matter hyperintensities, SLI: silent lacunar infarct

## Discussion

In the present study we investigated the differential impact of serum T-bil on two distinct pathological conditions of cerebral vessels in the same subjects: cerebral atherosclerosis and SVD. We investigated multiple indicators of cerebral atherosclerosis and cerebral SVD by use of brain MRI and MR angiography. Although carotid ultrasonography and retinography are useful tools to detect carotid atherosclerosis and microangiopathy, respectively, MR evaluation has advantages over them to evaluate both disease conditions simultaneously. Brain MRI clearly shows the status of cerebral SVD, and MR angiography can show the status of multiple extracranial- and intracranial arteries.

Consistent with previous studies [9–13], we found that the serum T-bil level was negatively associated with cerebral atherosclerosis, especially with ECAS. After adjusting for confounding factors, subjects in the middle and high T-bil tertiles displayed a 37% and 46% lower prevalence of ECAS, respectively, compared to those in the low T-bil tertile. Based on a spline curve analysis, the risk of ECAS markedly increased as the serum T-bil level decreased. This finding support previous findings showing that a low serum T-bil was closely related to various indices of carotid atherosclerosis. Mildly elevated serum bilirubin levels were found to be negatively associated with several indices of carotid atherosclerosis, such as carotid intima-media thickness or carotid plaque [9–13].

Compared to a strong association between serum T-bil and ECAS, the association between serum T-bil and ICAS was marginal. The mechanism of the differential impact of serum T-bil on ECAS and ICAS is unclear. One possible explanation is differences in metabolic and biological properties between intracranial and extracranial arteries. It has been suggested that extracranial arteries are more vulnerable to dyslipidemia because atherogenic particles such as oxidized LDL cholesterol are able to be transported into vascular walls more easily because of the higher fluid residence time of extracranial as opposed to intracranial arteries [21]. Clinical observations [22–25] and our results have shown that dyslipidemia was more prevalent in cases with ECAS than those with ICAS. Bilirubin has been reported to eliminate reactive oxygen species and to inhibit the oxidation of LDL cholesterol [5]. This finding may explain why the serum T-bil level is strongly associated with ECAS compared to ICAS. Another explanation is the heterogeneous pathological nature of ICAS. A recent study, based on high resolution MRI, showed that a significant proportion of cases who initially were diagnosed with presumed atherosclerosis with conventional MR angiography actually had a non-atherosclerotic vascular stenosis, such as atypical unilateral moyamoya disease, or intracranial arterial dissection [26, 27].

Another finding of our study is that MR indices of cerebral SVD were not associated with the serum T-bil level. Few studies, to date, have been performed within the context of observing an association between serum bilirubin and cerebral SVD. A cross-sectional study in Chinese population showed that subjects with a low serum T-bil level showed a high prevalence of silent cerebral infarct after adjusting for potential confounders [14]. Other cross-sectional study in Korean population revealed that the prevalence of cerebral WMH was 5.5-fold higher in women within a low serum T-bil tertile than in those within a high serum T-bil tertile [15]. However, we did not find a similar association. Reasons for discrepancies between studies may be due to differences in study design and subject characteristics. In addition, the definition of silent brain infarct (infarct of 3mm or more in diameter) [14] and WMH (presence of WMH lesion without the use of visual scale) [15] in previous studies may produce different results from our study. Specifically, the previous studies did not evaluate the status of cerebral atherosclerosis [14, 15], which is a strong predictor of the serum T-bil level in our study. For the clear association between serum bilirubin and cerebral SVD, we suggest that co-evaluation of cerebral atherosclerosis and SVD are required in the further studies.

Our study showed several limitations. First, as this study was a retrospective design, a selection bias was present. Furthermore, study subjects visited the outpatient department seeking medical attention for underlying cardiovascular risk factors, and thus, their demographic characteristics were not identical with those of the general population. Second, we measured the total form of serum bilirubin only. Since indirect bilirubin is a free form and is known to have an anti-oxidant effect, indirect bilirubin should be evaluated to reach a clearer conclusion concerning the relationship between the serum T-bil level and cerebral atherosclerosis. However, most previous studies measured the T-bil level, which enables us to compare the results of our study with these studies. Third, T-bil level may also be affected by diseases other than cerebral vascular disease, such as peripheral arterial occlusive disease [8] and diabetic neuropathy [28]. We did not investigate such conditions that could affect our outcomes. Finally, it is not immediately clear whether a low serum T-bil level is a causative factor or a consequence of atherosclerosis in our present study. Therefore, a prospective observation conducted on the general population is required to validate our results.

## Conclusions

We found a negative association between the serum T-bil level and cerebral atherosclerosis, especially extracranial arterial stenosis. On the other hand, the serum T-bil level was not associated with MR indices of SVD. Although further studies are needed, our results provide strong evidence of serum T-bil as a marker of cerebral atherosclerosis.

## Supporting information

S1 Table File containing patient information. (XLS)

## References

[1] Amarenco P, Bogousslavsky J, Caplan LR, Donnan GA, Hennerici MG. Classification of stroke subtypes. Cerebrovascular diseases (Basel, Switzerland). 2009;27(5):493–501. 10.1159/000210432 19342825

[2] Fisher CM. The arterial lesions underlying lacunes. Acta neuropathologica. 1968;12(1):1–15. 5708546 10.1007/BF00685305

[3] Hassan A, Hunt BJ, O'Sullivan M, Parmar K, Bamford JM, Briley D, et al Markers of endothelial dysfunction in lacunar infarction and ischaemic leukoaraiosis. Brain: a journal of neurology. 2003;126(Pt 2):424–32. 12538408 10.1093/brain/awg040

[4] Wardlaw JM, Farrall A, Armitage PA, Carpenter T, Chappell F, Doubal F, et al Changes in background blood-brain barrier integrity between lacunar and cortical ischemic stroke subtypes. Stroke; a journal of cerebral circulation. 2008;39(4):1327–32. 10.1161/STROKEAHA.107.500124 18309161

[5] Stocker R, Yamamoto Y, McDonagh AF, Glazer AN, Ames BN. Bilirubin is an antioxidant of possible physiological importance. Science (New York, NY). 1987;235(4792):1043–6. 10.1126/science.3029864 3029864

[6] Baranano DE, Rao M, Ferris CD, Snyder SH. Biliverdin reductase: a major physiologic cytoprotectant. Proceedings of the National Academy of Sciences of the United States of America. 2002;99(25):16093–8. 10.1073/pnas.252626999 12456881 PMC138570

[7] Akboga MK, Canpolat U, Sahinarslan A, Alsancak Y, Nurkoc S, Aras D, et al Association of serum total bilirubin level with severity of coronary atherosclerosis is linked to systemic inflammation. Atherosclerosis. 2015;240(1):110–4. 10.1016/j.atherosclerosis.2015.02.051 25770689

[8] Perlstein TS, Pande RL, Beckman JA, Creager MA. Serum total bilirubin level and prevalent lower-extremity peripheral arterial disease: National Health and Nutrition Examination Survey (NHANES) 1999 to 2004. Arteriosclerosis, thrombosis, and vascular biology. 2008;28(1):166–72. 10.1161/ATVBAHA.107.153262 17975120

[9] Erdogan D, Gullu H, Yildirim E, Tok D, Kirbas I, Ciftci O, et al Low serum bilirubin levels are independently and inversely related to impaired flow-mediated vasodilation and increased carotid intima-media thickness in both men and women. Atherosclerosis. 2006;184(2):431–7. 10.1016/j.atherosclerosis.2005.05.011 15979081

[10] Ishizaka N, Ishizaka Y, Takahashi E, Yamakado M, Hashimoto H. High serum bilirubin level is inversely associated with the presence of carotid plaque. Stroke; a journal of cerebral circulation. 2001;32(2):580–3. 10.1161/01.str.32.2.580-b 11157203

[11] Kawamoto R, Ninomiya D, Hasegawa Y, Kasai Y, Kusunoki T, Ohtsuka N, et al Mildly elevated serum bilirubin levels are negatively associated with carotid atherosclerosis among elderly persons. PloS one. 2014;9(12):e114281 10.1371/journal.pone.0114281 25479598 PMC4257609

[12] Yang XF, Chen YZ, Su JL, Wang FY, Wang LX. Relationship between serum bilirubin and carotid atherosclerosis in hypertensive patients. Internal medicine (Tokyo, Japan). 2009;48(18):1595–9. 10.2169/internalmedicine.48.2286 19755760

[13] Kawamoto R, Ninomiya D, Hasegawa Y, Kasai Y, Kusunoki T, Ohtsuka N, et al Mildly elevated serum total bilirubin levels are negatively associated with carotid atherosclerosis among elderly persons with type 2 diabetes. Clin Exp Hypertens. 2016;38(1):107–12. 10.3109/10641963.2015.1060990 26362780

[14] Li RY, Cao ZG, Zhang JR, Li Y, Wang RT. Decreased serum bilirubin is associated with silent cerebral infarction. Arteriosclerosis, thrombosis, and vascular biology. 2014;34(4):946–51. 10.1161/ATVBAHA.113.303003 24371085

[15] Park BJ, Shim JY, Lee HR, Kang HT, Lee JH, Lee YJ. Association between serum total bilirubin level and leukoaraiosis in Korean adults. Clinical biochemistry. 2012;45(4–5):289–92. 10.1016/j.clinbiochem.2011.12.023 22245549

[16] Lee HB, Kim J, Kim SH, Kim S, Kim OJ, Oh SH. Association between serum alkaline phosphatase level and cerebral small vessel disease. PLoS One 2015;10(11):e0143355 10.1371/journal.pone.0143355 26580067 PMC4651565

[17] Clase CM, Garg AX, Kiberd BA. Prevalence of low glomerular filtration rate in nondiabetic Americans: Third National Health and Nutrition Examination Survey (NHANES III). Journal of the American Society of Nephrology: JASN. 2002;13(5):1338–49. 11961022 10.1097/01.asn.0000013291.78621.26

[18] Fisher M, Martin A, Cosgrove M, Norris JW. The NASCET-ACAS plaque project. North American Symptomatic Carotid Endarterectomy Trial. Asymptomatic Carotid Atherosclerosis Study. Stroke; a journal of cerebral circulation. 1993;24(12 Suppl):I24–5; discussion I31-2. 8249015

[19] Chimowitz MI, Lynn MJ, Howlett-Smith H, Stern BJ, Hertzberg VS, Frankel MR, et al Comparison of warfarin and aspirin for symptomatic intracranial arterial stenosis. The New England journal of medicine. 2005;352(13):1305–16. 10.1056/NEJMoa043033 15800226

[20] Fazekas F, Chawluk JB, Alavi A, Hurtig HI, Zimmerman RA. MR signal abnormalities at 1.5 T in Alzheimer's dementia and normal aging. AJR American journal of roentgenology. 1987;149(2):351–6. 10.2214/ajr.149.2.351 3496763

[21] Aboyans V, Lacroix P, Criqui MH. Large and small vessels atherosclerosis: similarities and differences. Progress in cardiovascular diseases. 2007;50(2):112–25. 10.1016/j.pcad.2007.04.001 17765473

[22] Kim JS, Nah HW, Park SM, Kim SK, Cho KH, Lee J, et al Risk factors and stroke mechanisms in atherosclerotic stroke: intracranial compared with extracranial and anterior compared with posterior circulation disease. Stroke; a journal of cerebral circulation. 2012;43(12):3313–8. 10.1161/STROKEAHA.112.658500 23160885

[23] Kim YD, Choi HY, Jung YH, Nam CM, Yang JH, Cho HJ, et al Classic risk factors for atherosclerosis are not major determinants for location of extracranial or intracranial cerebral atherosclerosis. Neuroepidemiology. 2009;32(3):201–7. 10.1159/000195690 19169042

[24] Kuller L, Reisler DM. An explanation for variations in distribution of stroke and arteriosclerotic heart disease among populations and racial groups. American journal of epidemiology. 1971;93(1):1–9. 4924090 10.1093/oxfordjournals.aje.a121223

[25] Caplan LR, Gorelick PB, Hier DB. Race, sex and occlusive cerebrovascular disease: a review. Stroke; a journal of cerebral circulation. 1986;17(4):648–55. 10.1161/01.str.17.4.648 3526645

[26] Kwak JH, Choi JW, Park HJ, Chae EY, Park ES, Lee DH, et al Cerebral artery dissection: spectrum of clinical presentations related to angiographic findings. Neurointervention. 2011;6(2):78–83. 10.5469/neuroint.2011.6.2.78 22125753 PMC3214819

[27] Kim JS, Bonovich D. Research on intracranial atherosclerosis from the East and west: why are the results different? Journal of stroke. 2014;16(3):105–13. 10.5853/jos.2014.16.3.105 25328869 PMC4200588

[28] Kim ES, Lee SW, Mo EY, Moon SD, Han JH. Inverse association between serum total bilirubin levels and diabetic peripheral neuropathy in patients with type 2 diabetes. Endocrine. 2015 11;50(2):405–12. 10.1007/s12020-015-0583-0 25846483

